# The Physiological Function of nNOS-Associated CAPON Proteins and the Roles of CAPON in Diseases

**DOI:** 10.3390/ijms242115808

**Published:** 2023-10-31

**Authors:** Wenshuo Xie, Nianhong Xing, Jicheng Qu, Dongwu Liu, Qiuxiang Pang

**Affiliations:** Anti-Aging & Regenerative Medicine Research Institution, School of Life Sciences and Medicine, Shandong University of Technology, Zibo 255049, China; 22410010008@stumail.sdut.edu.cn (W.X.); 21410010894@stumail.sdut.edu.cn (N.X.);

**Keywords:** neuronal-type nitric oxide synthase, nitric oxide, carboxy-terminal PDZ ligand of neuronal nitric oxide synthase, physiological function, disease

## Abstract

In this review, the structure, isoform, and physiological role of the carboxy-terminal PDZ ligand of neuronal nitric oxide synthase (CAPON) are summarized. There are three isoforms of CAPON in humans, including long CAPON protein (CAPON-L), short CAPON protein (CAPON-S), and CAPON-S’ protein. CAPON-L includes three functional regions: a C-terminal PDZ-binding motif, carboxypeptidase (CPE)-binding region, and N-terminal phosphotyrosine (PTB) structural domain. Both CAPON-S and CAPON-S’ only contain the C-terminal PDZ-binding motif. The C-terminal PDZ-binding motif of CAPON can bind with neuronal nitric oxide synthase (nNOS) and participates in regulating NO production and neuronal development. An overview is given on the relationship between CAPON and heart diseases, diabetes, psychiatric disorders, and tumors. This review will clarify future research directions on the signal pathways related to CAPON, which will be helpful for studying the regulatory mechanism of CAPON. CAPON may be used as a drug target, which will provide new ideas and solutions for treating human diseases.

## 1. Introduction

In animals, the function of genes is mainly achieved through proteins. The function of proteins is related to amino acid sequences and protein folding patterns. Post-translational modifications also play a role in the development of diseases. As an important pattern of post-translational modification, the S-nitrosylation of proteins is associated with endoplasmic reticulum stress [1], cellular autophagy and apoptosis [1], vascular endothelial tension [2], energy metabolism and mitosis [3,4,5], cardiovascular diseases [2,3,6], and neurological diseases in humans [7,8]. Nitric oxide (NO) is one of the key molecules involved in protein S-nitrosylation [8,9]. NO is synthesized by nitric oxide synthase (NOS), which is a biologically active molecule in the human body. NO is produced from L-arginine via the catalysis of NOS and cofactors [8,10,11], and it further combines with cysteine thiols or sulfhydryl groups of target proteins and finally forms S-nitrosothiol derivatives [12,13,14]. There are three types of NOS, including neuronal-type nitric oxide synthase (nNOS), inducible-type nitric oxide synthase (iNOS), and endothelial-type nitric oxide synthase (eNOS). Both nNOS and iNOS are dependent on calcium, but eNOS is not dependent on calcium to play physiological functions [10,15,16]. The three types of NOS mediate various pathophysiological events in the cerebellum, skeletal muscles, kidneys, blood vessels, islets, neutrophils, and skin [17,18,19,20,21,22]. There are mainly nine proteins that can interact and bind with nNOS [23], among which the carboxy-terminal PDZ ligand of nNOS (CAPON) plays a key role in regulating the activity of nNOS [24]. CAPON participates in regulating degenerative neurological diseases [25,26], neurotoxicity [27], heart diseases, and various other diseases [28]. CAPON can be used as a drug target, which brings new ideas for treating human diseases. In this review, we will summarize the structure, isoform, and physiological role of CAPON. The relationship between CAPON and human diseases is introduced, which will provide an outlook for future studies on the diseases related to CAPON.

## 2. Definition and Subtypes of CAPON

CAPON has a C-terminal PDZ-binding motif, which can bind and interact with the N-terminal PDZ domain of nNOS [29]. Thus, it is named the carboxy-terminal PDZ ligand of nNOS [30], and it is known as an adaptor protein of nNOS [31,32]. CAPON is a highly conserved protein and the conserved domain of CAPON includes 80~100 amino acid residues [30]. In the conserved domain of rat CAPON, 92% of amino acid sequences are consistent with human CAPON [30,33,34]. CAPON was first identified in the neuronal tissue of the rat brain [33], which participates in regulating the growth of dendrites and synapses [25]. It was later found that CAPON is present in the human brain, mouse heart [21], pancreas [35], and skeletal muscle [36]. Moreover, CAPON is abundant in the olfactory bulb, cerebellum, and hippocampus [33]. 

**Figure 1:**
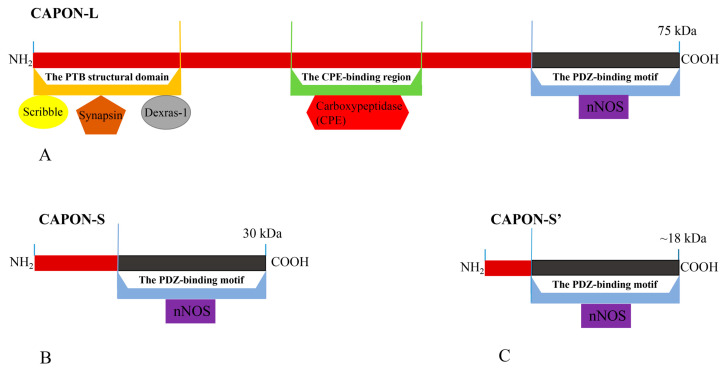
The structure and three isoforms of CAPON. (**A**) CAPON-L; (**B**) CAPON-S; (**C**) CAPON-S’. The differences in isoform length are indicated in red. CAPON-L contains three functional regions, but CAPON-S and CAPON-S’ only contain C-terminal PDZ-binding motif.

There are three isoforms of CAPON in humans, which include the long CAPON protein (CAPON-L), short CAPON protein (CAPON-S), and CAPON-S’ protein [37,38,39,40] (Figure 1). The CAPON-L protein (75 kDa) includes three functional regions: (1) C-terminal PDZ-binding motif; (2) carboxypeptidase (CPE)-binding region; and (3) N-terminal phosphotyrosine (PTB) structural domain [40] (Figure 1). The three functional regions of CAPON form a complete CAPON-L protein [39]. The molecular weight of the CAPON-S protein is 30 kDa [40], and the molecular weight of the CAPON-S’ protein is about 18 kDa [38] (Figure 1). Both CAPON-S and CAPON-S’ only contain the C-terminal PDZ-binding motif, which is a truncated version of CAPON-L. CAPON-S and CAPON-S’ may be formed by post-translational phosphorylation modification of CAPON. It has been found that CAPON-S and CAPON-S’ are present in the dorsolateral prefrontal cortex of the human brain and rat glioma [37,40]. The length of CAPON-S’ is less than that of CAPON-S due to its the shorter N-terminal domain [38,41] (Figure 1). 

## 3. Roles of Different Structural Domains of CAPON

### 3.1. The PDZ-Binding Motif at the C-Terminus of CAPON Binds with nNOS and Regulates NO Production

**Figure 2:**
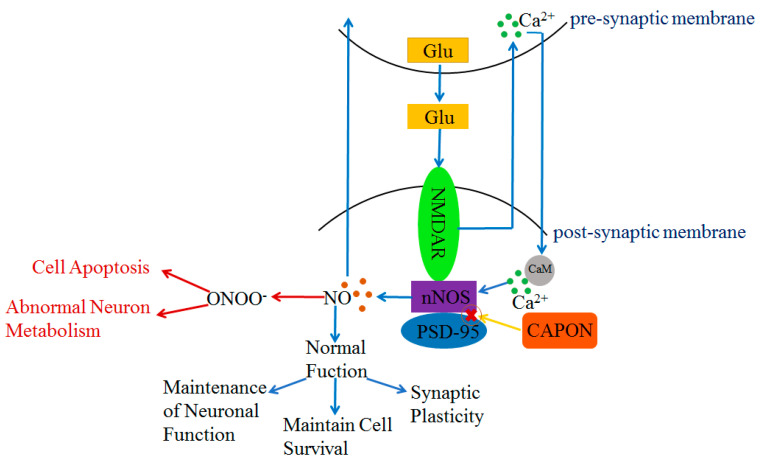
Regulation of NO content and NO function by NMDAR-nNOS-PSD-95/CAPON ternary complex. Blue arrows indicate that PSD-95 protein regulates NO content and controls normal physiological functions under normal conditions. Red arrows indicate PSD-95 is over-activated with excessive release of NO under abnormal physiological conditions, whereas CAPON can compete with PSD-95 to bind with nNOS to attenuate this process.

The PDZ structural domain of nNOS plays a key role in interacting with other proteins. Both CAPON and postsynaptic density protein 95 (PSD-95) can bind with nNOS via the PDZ-binding motif [33,42,43]. Moreover, CAPON can compete with PSD-95 to bind with nNOS; CAPON disrupts the combination of PSD-95 and nNOS [33,44]. In the central nervous system (CNS), nNOS is present in the post-synaptic membrane [45,46]. N-methyl-D-aspartate receptor (NMDAR) is an ionotropic glutamate receptor [47,48,49], and nNOS is an important target protein in the downstream of NMDAR [50] (Figure 2). Once glutamate is released into the synaptic gap by the pre-synaptic membrane, glutamate will bind with NMDAR in the post-synaptic membrane (Figure 2). Subsequently, Ca^2+^ is released from the pre-synaptic membrane and binds with calmodulin (CaM) in the post-synaptic membrane [45,48,51] (Figure 2). Then, PSD-95 shifts from the cytoplasm to the plasma membrane [48] and binds with the NR_2_ subunit of NMDAR and nNOS to form a triplex complex NMDAR-nNOS-PSD-95 [30,52,53] (Figure 2). The formation of NMDAR-nNOS-PSD-95 leads to a mild phosphorylation of nNOS, and the activation of nNOS generates NO, which interacts with guanylate cyclase (sGC), intracellular proteins, and ion channels (Figure 2). The action of sGC generates cyclic guanosine monophosphate (cGMP). Both NO and cGMP further mediate the downstream signal pathways [45,54] (Figure 2). The triplet complex NMDAR-PSD95-nNOS plays an important role in a range of normal neuronal functions such as maintaining synaptic plasticity and cell survival, which has a positive effect on learning and memory [45,55,56,57]. NO also acts at the pre-synaptic membrane to regulate the release of neurotransmitters [48,54]. However, as NMDAR is overstimulated by excessive glutamate, it will produce excessive Ca^2+^ flowing inward to the post-synaptic membrane. Excessive inward Ca^2+^ not only affects the function of post-synaptic neurons, but also causes the over-phosphorylation of nNOS by the triplet complex NMDAR-PSD95-nNOS. The over-activation of nNOS may result in the over-production of NO [51]. Excessive NO acts with superoxide ions (O_2_^·-^) and forms peroxynitrite (ONOO^-^) [58], which causes disorders of neuronal metabolism and triggers cell apoptosis [59] (Figure 2). In contrast, CAPON can compete with PSD-95 to bind with nNOS and disrupts the triplet complex NMDAR-PSD95-nNOS. The new complex NMDAR-CAPON-nNOS attenuates the activity of nNOS and reduces the amount of NO. The formation of NMDAR-CAPON-nNOS plays a key role in protecting neuronal metabolism and function by decreasing the over-activation of nNOS (Figure 2). The moderate formation of PSD95-nNOS has a positive effect on synaptic connections. The over-expression of CAPON inhibits the moderate formation of NMDAR-PSD95-nNOS, which may cause damage to the nervous system [60].

### 3.2. The Carboxypeptidase (CPE)-Binding Region of CAPON Inhibits Neuronal Dendrite Growth

The carboxypeptidase (CPE)-binding region of CAPON-L includes 127 amino acids at sites 181–307, which are the intermediate region of CAPON-L [34,39]. Carrel et al. found that the overexpression of CAPON-L reduced the number of neuronal dendrites, but the interference of CAPON-L increased the number of neuronal dendrites in the embryonic hippocampus of rats [39]. The reason is that the CPE-binding region of CAPON-L directly binds with CPE, and the interaction of this complex reduces the number of dendritic branches and inhibits neurite growth [39]. CAPON-S only has a PDZ-binding motif but no CPE-binding region. Thus, CAPON-S cannot regulate the number of dendritic branches and neurite growth. It has been confirmed that neither uncoupling nNOS-CAPON interactions nor increasing the N-terminal PTB structural domain of CAPON affects the growth of dendritic branches [61]. It can be concluded that the CPE-binding region in the middle region of CAPON-L is important for dendrite growth. In addition, Crosta et al. found that overexpression of CAPON-L may play a long-term role in different developmental stages of dendritic formation, growth, and maintenance, and it significantly reduces the number of primary and secondary dendrites [62]. CAPON-S plays a short-term and limited role in regulating dendritic branching, and it only affects early dendritic growth and branch development [62]. 

### 3.3. The N-Terminal PTB Structural Domain of CAPON Binds to a Variety of Bridging Proteins to Regulate Different Physiological Functions

**Figure 3:**
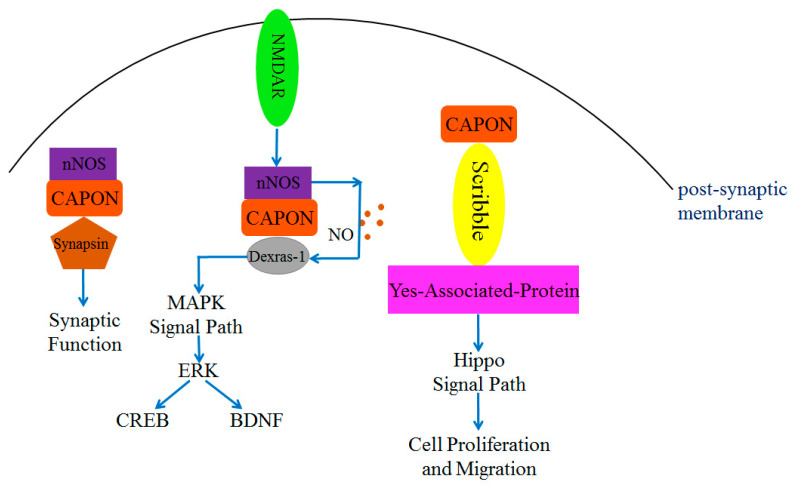
CAPON-related complex proteins regulate MAPK signal pathway, synaptic function, and Hippo signal pathway. Both Scribble and Synapsin can directly bind with CAPON and induce downstream signals to regulate physiological functions. The nitrosylation of Dexras1 requires NO released from nNOS to regulate downstream pathways.

The N-terminal PTB structural domain of CAPON can bind and function with multiple proteins, such as Scribble, the Synapsin family (including Synapsin I, Synapsin II, and Synapsin III), and Dexras1 [30]. Scribble is a cell polarity protein that is present in the pre-synaptic and post-synaptic membranes [63,64], and it exists as a scaffolding protein. Scribble directly connects with CAPON without the assistance of other proteins. This connection occurs through the involvement of the PTB structural domain of CAPON and the PDZ-binding motif of Scribble [63], which participates in regulating neuronal growth [48,63,65]. Overexpression of CAPON affects the number of dendrites and synaptic spinogenesis in the presence of a PTB structural domain [63]. Thus, it can be concluded that CAPON is essential for synapse growth in mammals [48]. In addition, Scribble plays a role in cell polarity, differentiation, and migration [37,64,66]. It also functions in tumorigenesis inhibition, and it can bind with Yes-associated protein (YAP) and connect with CAPON to form a triplet complex CAPON-Scribble-YAP [67] (Figure 3). YAP is one of the molecules in the Hippo signaling pathway and plays a key role in cell proliferation and differentiation [68]. YAP is involved in tumor proliferation and migration [37,41,69]. Therefore, CAPON may affect cell proliferation and migration through the Scribble or Hippo signaling pathways (Figure 3).

The PTB structural domain of CAPON connects with a signaling pathway that is associated with Dexras1. Dexras1 is a small molecule G protein mainly expressed in the brain [70,71]. It binds with NMDAR receptors and is toxic to neurons [48,70]. Dexras1 may exert toxicity to neurons in the central nervous system by regulating NO-related signaling pathways [65,72,73] (Figure 2). The N-terminal PTB structural domain of CAPON binds with Dexras1 and forms a duplex complex. Subsequently, nNOS is activated by NMDAR from the post-synaptic membrane, and nNOS could bind with the PDZ-binding motif of CAPON and form a triplet complex nNOS-CAPON-Dexras1 [65,72]. The interaction of nNOS and CAPON generates NO and acts on Dexras1, which will lead to Cys11 S-nitrosylation in Dexras1 [34]. The binding of CAPON and nNOS enhances the activity of nNOS and NO production [25,34] (Figure 3).

Dexras1 is also a member of the RAS superfamily, and it is associated with the mitogen-activated protein kinase (MAPK) signaling pathway [25,74]. The nitrosylation of Dexras1 further regulates a downstream extracellular signal-regulated kinase (ERK), which is involved in regulating synaptic growth and emotional behavior [75,76]. As Dexras1 is overexpressed, the phosphorylation level of ERK will be reduced, which in turn reduces the phosphorylation level of downstream cyclic adenosine monophosphate (cAMP) response element binding protein (CREB) and the expression level of brain-derived neurotrophic factor (BDNF) [57]. This negative regulatory function of Dexras1 has been observed in the brain [75,76]. Dexras1 regulates NO-related signaling pathways by forming a triplet complex with nNOS and CAPON, which may cause neuronal cell death in the CNS [45,72] (Figure 3). Since a total of 180 N-terminal amino acids of CAPON have been proven to contain the structural domains required for binding to Scribble, Dexras1, and Synapsin proteins, it is possible that only CAPON-L can serve as a bridging protein to connect nNOS with these target proteins [30,34]. The role of CAPON-S may competitively inhibit the binding of other ligands with PDZ domains of nNOS and PSD93/PSD95 [40].

## 4. CAPON and Human Diseases

### 4.1. CAPON Induces Cardiac Diseases by Regulating Ca^2+^ Channel

Congenital long QT syndrome (LQTS) is an inherited disease that results from abnormal cardiomyocyte repolarization. An abnormally prolonged QT interval causes cardiac rhythm abnormalities, which may consequently threaten life [28,77,78]. The reason is usually related to variants in certain ion channel subunits that are involved in regulating cardiac action potential [79]. The mutation of pore subunits which encode potassium channels is the most common cause of LQTS [80,81]. Previously, some studies have indicated that different variants of *capon* contribute to the development of this disease. Several variants of *capon* (rs10494366, rs4657139, and rs6683868) in a community population were confirmed to be associated with a longer QT interval in the carriers of these mutations [21,82]. In South Africa, the *capon* variants in the LQTS population included rs4657139 and rs16847548 [32,79]. These *capon* variants resulted in a prolonged QT interval, which are associated with diseases such as sudden cardiac death (SCD) and cardiac arrest [79]. Chang et al. found that overexpression of CAPON interacted with nNOS to produce NO in isolated guinea pig myocytes [83,84]. It further inhibited type I calcium currents via S-nitrosylation and enhanced delayed rectifier currents, which would lead to shortened action potentials [17,79]. If the interaction between CAPON and nNOS in cardiac myocytes is reduced, the decrease in NO content causes an increase in type I calcium currents and a decrease in delayed rectifier K^+^ currents. It could lead to a prolongation of action potential and QT interval, which will increase the risk of sudden cardiac death. Most SCDs occur in the context of coronary artery disease, but this still needs to be further validated [32,85]. In a single-family Saudi clinical and genetic analysis, the identified *capon* variant (rs4657139) did not affect QT interval [86]. This result indicates that single-nucleotide polymorphisms (SNPs) of *capon* are different among different ethnicities [32]. Tobin et al. found that different *capon* variants were responsible for the duration of QT interval prolongation between genders [87] (Table 1). Monique et al. reached the opposite conclusion when they studied the relationship between CAPON overexpression and the QT interval [88]. Their results showed that the overexpression of CAPON in transgenic mice resulted in the nitrosylation of the cardiac subtype of voltage-gated type I calcium channels, and it decreased the action potential of APD90 and the QT interval [88]. With the development of biological technology, the mechanism of CAPON and the QT interval needs to be further studied in future.

### 4.2. CAPON Induces Diabetes through Modulation of Ca^2+^-Related Signaling Pathways

The prolongation of the QT interval is not only associated with cardiac diseases but also associated with type I and type II diabetes [89] (Table 1). Lehtinen et al. demonstrated that the *capon* variants rs10494366 and rs10918594 were associated with the prolongation of the QT interval in families with genetic susceptibility to diabetes, which also explains the effect of diabetes on cardiac repolarization [90]. Becker et al. studied the relationship between *capon* and diabetes incidence by using calcium antagonists in patients with the *capon* variant rs10494366. The results showed that patients with the TT genotype were more vulnerable to diabetes than the patients with genotypes GG and TG, suggesting that the CAPON gene possibly affects diabetes via the Ca^2+^ signal [91]. Kaida et al. overexpressed *capon* in the human and mouse liver. Overexpression of CAPON improved insulin sensitivity via the C-terminal binding motif, but downregulated the phosphorylation of p38 MAPK [92]. The result of Kaida et al. demonstrates the key role of CAPON in the development of type II diabetes. Zhao et al. found that nNOS activity was positively correlated with hepatic insulin resistance. Higher nNOS activity leads to lower insulin sensitivity and upregulates the phosphorylation level of p38 MAPK. nNOS expression in the liver is negatively correlated with CAPON level and decreases p38 MAPK activity [93]. Therefore, CAPON may act through the inactivation of nNOS in liver (Table 1).

### 4.3. CAPON Affects Neuronal and Dendritic Spine Development in Brain

Overexpression of CAPON not only affects the development of neurons and the nervous system but also causes mitochondrial dysfunction. Most diseases are related to the NMDAR-mediated CAPON-nNOS pathway [94], and CAPON may be a drug target for various clinical psychiatric disorders [41]. Acute injury of the cerebrospinal cord is a common traumatic disease. In rat models with spinal cord or sciatic nerve injury, an increase in CAPON-nNOS enhanced the expression level of Dexras1 [95,96]. It has been shown that CAPON causes acute brain injury through the P38 MAPK pathway, which is associated with the C-terminal binding motif of CAPON [27]. Abnormal increases in CAPON also contribute to this disease [97,98]. Gu et al. found that attenuated nNOS–CAPON interactions reduced dendritic complexity and promoted functional recovery after stroke [99]. Li et al. developed a cell-permeable peptide that interacts with CAPON–nNOS; this peptide could decrease excitotoxicity and damage in animal models with neonatal brain hypoxia-ischemia [27]. Thus, CAPON may be a drug target for treating ischemic stroke disease (Table 1).

Neurodegenerative diseases mainly include Alzheimer’s disease [25,57], Huntington’s disease, Parkinson’s disease [100], amyotrophic lateral sclerosis [101], and mood disorders such as major depression [102], bipolar disorder [102], anxiety disorders [103] and post-traumatic stress disorder (PTSD) [104,105]. These diseases are associated with the interaction of nNOS and CAPON. Overexpression of CAPON causes pathological Tau protein phosphorylation. It results in the activation of the triplet complex Dexras1-nNOS-CAPON, which will lead to β-amyloid deposition and aggravate Alzheimer’s disease. Under certain circumstances, it even leads to neuronal death and synaptic dysfunction [94,106]. It also affects the downstream MAPK signal pathway and some other signal pathways, which causes a decrease in ERK phosphorylation and P38 activation and finally leads to neurotoxicity [25]. Shi et al. investigated the effect and role of the dissociation of hippocampal dentate gyrus (DG)-nNOS-CAPON on the anxiolytic and antidepressant effects of fluoxetine. They found that the conjunction of CAPON–nNOS affects neuroplasticity and CAPON–nNOS reduces the phosphorylation levels of ERK, CREB, and BDNF. A lower phosphorylation level of these proteins mediates neuroplasticity pathways, which leads to an increase in depressive and anxiety-like behaviors [107]. Thus, they are a potential drug target for the treatment of psychiatric and neurological disorders (Table 1).

Schizophrenia is a genetic disorder which results in genetic defects [108,109]. Brzustowicz et al. analyzed 15 SNPs of *capon* in Canadian families, and eventually found three SNPs associated with schizophrenia [110]. Dilhan et al. found that the interaction of two *capon* SNPs (rs12143842 and rs10494366) and antipsychotic drugs affected the QT interval in patients, and this effect was related to gender. Males with both SNPs on the major allele had a progressive prolongation of the QT interval with increasing drug concentrations during the administration of antipsychotic medication [111]. However, this significant interaction effect was not found in female patients [111]. Xu et al. found that the mRNA level of CAPON-S was significantly elevated in the brains of patients with schizophrenia by analyzing post-mortem brain tissue, but the mRNA level of CAPON-L remained unchanged [40]. Antipsychotic drug therapy seems to have no regulatory effect on the mRNA expression level of CAPON-S. In bipolar disorder, the expression level of CAPON-S is not significantly correlated with a lifetime history of antipsychotic drug use and a history of antipsychotic drug use at death. Carrel et al. demonstrated that NOS1AP-S mRNA is not affected by antipsychotic drugs. They also found that the expression level of NOS1AP-S mRNA was significantly higher in the tissues of patients with schizophrenia and bipolar disorder compared to the control group [39]. This discovery also supports the viewpoint of Xu et al. Therefore, CAPON-S may be closely related to schizophrenia and bipolar disorder (Table 1).

### 4.4. CAPON Affects Tumor Growth

**Figure 4:**
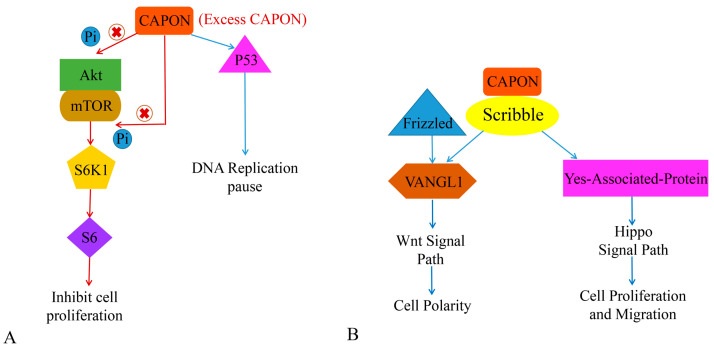
The role of CAPON in tumor growth. (**A**) CAPON inhibits cell proliferation via Akt/mTOR signaling pathway and induces DNA replication pause via P53. (**B**) CAPON induces cell polarity, cell proliferation, and cell migration via VANGL1/Wnt and Hippo signaling pathways.

Both CAPON-S and CAPON-L have the function of regulating cell proliferation [37]. The combination of CAPON and Scribble affects cellular proliferation and migration. The reduced expression level of CAPON enhances the growth and survival of certain cells that share the feature of being able to grow independently without cell anchoring. However, overexpression of CAPON-S inhibits the growth of non-tumor cells but induces the growth of tumor cells [67]. Gao et al. demonstrated that the overexpression of CAPON-S inhibits the proliferation of glioma cells, arrests the glioma cell cycle in G1 phase, and inhibits the serine/threonine kinase (Akt)-S6 ribosomal protein cell signaling pathway (Akt-S6 cell signaling pathway). In addition, it is frequently activated in glioma cells without affecting the expression level of signaling molecules related to the MAPK signaling pathway [37,67]. Liang et al. found that CAPON-L overexpression affects the Akt-mTOR-S6 signaling pathway in myeloma U251 cells, which in turn inhibits U251 cell proliferation (Figure 4A). However, this effect is not present in U87 cells, while overexpression of CAPON-S inhibits Akt signaling in U87 cells [112]. Shen et al. found that CAPON overexpression was associated with myeloma cell adhesion, cell growth, and cell-adhesion-mediated drug resistance [113]. A decrease in CAPON expression level not only induces the expression of Akt and p-Akt, but also increases cell adhesion and shortens the cellular G1 cycle. It also promotes cell growth and reduces the sensitivity of cells to chemotherapeutic drugs. However, the effect of CAPON on myeloma cells needs to be further investigated, which may provide new ideas for tumor treatment. Anastas et al. found that CAPON promotes the migration of breast cancer cells by conjunction with Scribble (SCRIB) and induces van-like protein-1 (VANGL1) [114]. VANGL1 may further induce the Wnt signal pathway [115] (Figure 4B). It has been shown that reduced CAPON expression levels in breast cancer decrease the proliferation of breast cancer cells [67,114]. Therefore, this evidence suggests that CAPON may be a potential oncogenic factor in tumors (Table 1).

### 4.5. CAPON and Skeletal Muscle Disease

Duchenne muscular dystrophy (DMD) is a genetic disorder which leads to severe muscle degeneration and dilated cardiomyopathy. The signal pathway related to NO can improve cardiac function and skeletal muscle in mdx mice, a mouse model for DMD [116]. It has been found that periodic acceleration (pGz) increases muscle force generation and expression of CAPON in mdx mice. The potential therapeutic efficacy of pGz may be presented as a non-pharmacological and non-invasive approach for treating DMD patients via the activation of the NO pathway [116]. In a previous study, CAPON was identified in mouse muscle by using Western and Northern blotting and in situ hybridization [36]. The *capon* gene was expressed in developing dystrophic and normal muscles near fiber junctions with tendons. In regenerating normal and dystrophic muscles in mdx mice, CAPON was prominent in satellite new myotubes and cells. The level of CAPON was increased in dystrophic quadriceps muscles after treatment with steroid deflazacort plus L-arginine [36]. The identification of CAPON in mammalian muscle showed that CAPON may have a functional role in stabilizing neuronal NOS in skeletal muscle, and it may be used to treat human muscular dystrophy [36].

**Table 1:** Diseases related to CAPON.

Disease Type	Symptoms of Disease	References
Heart disease	Congenital long QT syndrome	[21,32,82,87]
	Sudden cardiac death	[79,83,85]
	Cardiac arrest	[79]
Diabetes	Type 2 diabetes	[90,91,92,93]
Dendritic development of neurons in the brain	Acute brain injury	[27,97,98]
	Acute spinal cord injury	[99]
Neurodegenerative diseases	Alzheimer’s disease	[25,57]
	Huntington’s disease	[117,118]
	Parkinson’s disease	[100]
	Amyotrophic lateral sclerosis	[101]
	Major depressive disorder	[102,107]
	Bipolar disorder	[102]
	Anxiety disorders	[33,103,107]
	Post-traumatic stress disorder	[104,105]
Schizophrenia	Schizophrenia	[40,110,111]
Cerebrovascular disease	Ischemic stroke	[27,99]
Skeletal muscle disease	Duchenne muscular dystrophy	[36,116]
Cancer	Glioma	[67,112]
	Myeloma	[112,113]
	Breast cancer	[67,114]

## 5. CAPON and Disease Related to Genetic Association

Genetic factors strongly influence humans’ susceptibility to diseases. As each causal gene makes a small contribution to heritability, it is difficult to identify disease-related genes [119]. Genome-wide association studies are a powerful approach for mapping causal genes. Studies on genetic association will be a key tool for treating and understanding human diseases [119,120]. Some evidence supports the *capon* gene as a susceptibility gene for schizophrenia, but there is still a difference in the results of independent association studies on this gene. Single-nucleotide polymorphisms (SNPs) are DNA sequence polymorphisms caused by variations in a single nucleotide at the genomic level. They are the most common form of heritable variation in humans, accounting for over 90% in all known polymorphisms. SNPs are widely present in the human genome, with an average of one SNP in every three hundred base pairs. SNPs may be caused by the conversion, reversal, insertion, or deletion of bases. Previously, 15 SNPs and 14 microsatellites from the 5.4 Mb region between D1S1653 and D1S1677 in Canadian familial schizophrenia pedigrees have been analyzed [110]. Significant evidence of linkage disequilibrium (LD) was found between schizophrenia and six SNPs and two microsatellites. The significant LD in schizophrenia falls in the genomic extent of the *capon* gene, and 1q22 is the locus of schizophrenia susceptibility. It highlights the potential role of *capon* in understanding schizophrenia [110]. Since the 1q22 region is a harbor candidate schizophrenia susceptibility gene, nine single SNPs (spanning 236 kb regions of *capon*) were analyzed in a Han Chinese population. A significant difference was observed in the SNP rs348624 [121]. The results showed that the *capon* gene might be a candidate susceptibility gene for schizophrenia. Due to the complex causes of diseases, the interactions between multiple genetic factors and environmental influences may play a greater role in susceptibility to diseases [119].

## 6. Conclusions

In conclusion, we summarize the structure and isoforms of CAPON. CAPON participates in regulating NO production and neuronal development by binding with nNOS. An overview of the relationship between CAPON and heart diseases, diabetes, psychiatric disorders, and cancer is provided. This review will clarify future research directions on signal pathways related to CAPON, which is helpful for studying the regulatory mechanism of CAPON. In addition, CAPON may be used as a drug target, which will provide new ideas and solutions for treating human diseases. 

## Data Availability

All the data in the article are available from the corresponding author upon reasonable request.

## Abbreviations

Akt	serine/threonine kinase
BDNF	brain-derived neurotrophic factor
CaM	calmodulin
cAMP	cyclic adenosine monophosphate
CAPON	carboxy-terminal PDZ ligand of neuronal nitric oxide synthase
CAPON-L	long CAPON protein
CAPON-S	short CAPON protein
cGMP	cyclic guanosine monophosphate
CNS	central nervous system
CPE	carboxypeptidase
CREB	cAMP-response element binding protein
eNOS	endothelial-type nitric oxide synthase
ERK	extracellular signal-regulated kinase
iNOS	inducible-type nitric oxide synthase
LQTS	long QT syndrome
MAPK	mitogen-activated protein kinase
NMDAR	N-methyl-D-aspartate receptor
nNOS	neuronal nitric oxide synthase
nNOS	nitric oxide synthase
NO	nitric oxide
O_2_^·-^	superoxide ions
PSD-95	post-synaptic density protein 95
PTB	N-terminal phosphotyrosine
PTSD	post-traumatic stress disorder
SCD	sudden cardiac death
SCRIB	scribble
sGC	guanylate cyclase
SNPs	single-nucleotide polymorphisms
VANGL1	van-like protein-1
YAP	yes-associated protein
